# “They made me feel like I mattered”: a qualitative study of how mobile crisis teams can support people experiencing homelessness

**DOI:** 10.1186/s12889-024-19596-2

**Published:** 2024-08-12

**Authors:** Megan McDaniel, Siva Sundaram, Deepa Manjanatha, Rachel Odes, Paige Lerman, Margaret A. Handley, Phillip O. Coffin, Janet J. Myers, Matthew L. Goldman

**Affiliations:** 1 San Francisco Department of Public Health, 1380 Howard Street, San Francisco, CA USA; 2 Western Psychiatric Institute and Clinic, University of Pittsburgh Medical Center, 3811 O’Hara Street, Pittsburgh, PA USA; 3 Department of Psychiatry and Behavioral Sciences, University of California, San Francisco (UCSF), 675 18th Street, San Francisco, CA USA; 4 San Diego State University/University of California, San Diego, Joint Doctoral Program in Clinical Psychology, San Diego State University, University of California, San Diego, 6363 Alvarado Court, Suite 103, San Diego, CA USA; 5 National Clinician Scholars Program, University of California, San Francisco, San Francisco, CA USA; 6 School of Nursing, University of Wisconsin-Madison, Madison, WI USA; 7 School of Public Health, UCSF - UC Berkeley Joint Medical Program (JMP), University of California, Berkeley, 2121 Berkeley Way, Berkeley, CA USA; 8 School of Medicine, UCSF - UC Berkeley Joint Medical Program (JMP), University of California, San Francisco, 533 Parnassus Avenue, San Francisco, San Francisco, CA USA; 9 Department of Epidemiology and Biostatistics, University of California, San Francisco, 550 16th Street, 2nd Floor, San Francisco, CA USA; 10 Department of Medicine, University of California, San Francisco, 513 Parnassus Street, Room S-224, San Francisco, CA USA; 11 UCSF Partnerships for Research in Implementation Science for Equity (PRISE) Center, 550 16th Street, 3rd Floor, San Francisco, CA USA

**Keywords:** Mobile crisis teams, Homelessness, Qualitative research, Social-ecological model

## Abstract

**Background:**

Mobile crisis teams (MCTs) can be important alternatives to emergency medical services or law enforcement for low-acuity 911 calls. MCTs address crises by de-escalating non-violent situations related to mental health or substance use disorders and concurrent social needs, which are common among people experiencing homelessness (PEH). We sought to explore how an MCT in one city served the needs and supported the long- and short-term goals of PEH who had recently received MCT services.

**Methods:**

We conducted 20 semi-structured interviews with service recipients of the Street Crisis Response Team, a new 911-dispatched MCT implemented in San Francisco in November 2020. In the weeks after their encounter, we interviewed respondents about their overall MCT experience and comparisons to similar services, including perceived facilitators and barriers to the respondent’s self-defined life goals. We analyzed interview transcripts with thematic analysis to capture salient themes emerging from the text and organized within a social-ecological model.

**Results:**

Nearly all respondents preferred the MCT model over traditional first responders, highlighting the team’s person-centered approach. Respondents described the MCT model as effectively addressing their most immediate needs (e.g., food), short-term relief from the demands of homelessness, acute mental health or substance use symptoms, and immediate emotional support. However, systemwide resource constraints limited the ability of the team to effectively address longer-term factors that drive crises, such as solutions to inadequate quality and capacity of current housing and healthcare systems and social services navigation.

**Conclusions:**

In this study, respondents perceived this MCT model as a desirable alternative to law enforcement and other first responders while satisfying immediate survival needs. To improve MCT’s effectiveness for PEH, these teams could collaborate with follow-up providers capable of linking clients to resources and services that can meet their long-term needs. However, these teams may not be able to meaningfully impact the longstanding and complex issues that precipitate crises among PEH in the absence of structural changes to upstream drivers of homelessness and fragmentation of care systems.

**Supplementary Information:**

The online version contains supplementary material available at 10.1186/s12889-024-19596-2.

## Background

To address longstanding gaps in services for people experiencing mental health crises, localities across the US have begun to implement specialized mobile crisis teams (MCTs) as alternatives to emergency medical services and law enforcement response [1, 2]. MCTs were developed in the 1970’s as a way to dispatch teams of clinicians with specialized crisis training focused on de-escalation, rapport building, brief interventions, risk assessment and triage to determine whether a higher level of care is necessary” [3, 4]. By providing a rapid behavioral health specialty response, MCTs aim to reduce potentially harmful or traumatic law enforcement interactions with vulnerable individuals in crisis and to divert individuals from costly, overcrowded hospital-based services by resolving crises in the field [5, 6]. Policies such as the enhanced federal match for Medicaid reimbursement of MCT services authorized in the 2021 American Rescue Plan Act will increase the development of MCT services across the United States [7]. While some studies have found MCTs to be effective in addressing crises in preventing unnecessary hospitalization or incarceration, the current evidence base of what populations are best served by MCT intervention is sparse, and no studies have characterized MCTs that specialize in serving people experiencing homelessness [8].

People experiencing homelessness (PEH) represent a population well-suited for MCT intervention, given their intersectional vulnerabilities to behavioral health crises and to adverse outcomes from other crisis response services [9, 10]. Compared to the housed population, PEH face increased risk factors for behavioral health crises, like underlying health conditions, substance use, and specific life stressors [11]. PEH also have poor access to routine medical and behavioral health services with correspondingly high utilization of emergency department care [12]. PEH at baseline experience high rates of law enforcement interactions due to increased visibility and survival behaviors such as sleeping in public, contributing to high lifetime incarceration rates and homelessness [9]. Emergency medical services and law enforcement responses may not sufficiently address the underlying drivers of crisis for PEH nor link them with routine care that could help prevent further crises [13]. These factors suggest that PEH comprises a vulnerable population that could significantly benefit from MCT intervention over traditional emergency response.

While MCTs can offer potential advantages, the evidence base for their effectiveness remains underdeveloped. Existing studies of MCTs typically examine broader system-focused measures like emergency care reutilization rates, yet rarely has research in this area characterized the perspectives of those served by MCTs [14]. To understand how MCTs can better meet the needs of PEH, we interviewed individuals who had recently received crisis response and post-crisis follow-up by a new MCT program. We sought to explore how these teams did or did not meaningfully support the respondents’ crisis needs and life goals across personal, relationship, community, and societal levels.

## Methods

### Study setting and MCT model

Recognizing the challenges faced by PEH and the potential benefits of MCTs, policymakers in San Francisco implemented the Street Crisis Response Team (SCRT) in November 2020. SCRT is a 911-dispatched MCT offering 24/7 response to behavioral health crises in public spaces. This program employed three types of providers: a paramedic, a behavioral health clinician, and a peer specialist with lived experience of mental health or substance use condition. During each crisis encounter, the MCT delivered crisis support and offered post-crisis follow-up from case managers at the San Francisco Department of Public Health’s Office of Coordinated Care. Crisis supports focused on addressing the service recipient’s immediate needs like assessing mental and physical health needs; providing emotional support, first-aid (e.g., bandages), food and water, blankets and clothing; arranging for or directly providing transportation to an emergency shelter or a crisis stabilization center; and sharing resource information for addressing short-term needs and linking them to follow-up services for addressing long-term needs. The SCRT model provides a specialized approach to assisting with immediate survival needs relevant to those experiencing homelessness or housing instability. Post-crisis follow-up aimed to connect individuals with services like short-term shelter, coordinated entry assessment for housing, and medical, behavioral health, and substance use care.

### Study sample and recruitment

We interviewed twenty adults who had received services from SCRT within the previous six months. Respondents were recruited primarily through a referral from the Office of Coordinated Care during follow-up encounters and by circulating flyers in community settings where PEH congregated. Those who provided contact information during referral were contacted immediately for outreach, and we attempted in-person visits for those lacking contact information. At the time of outreach, we explained the study purpose and interview procedures, assessed eligibility based on receipt of SCRT services within the past six months, and offered a $60 gift card as compensation for completing the interview.

### Procedures

Prospective participants completed informed consent for the research team to conduct the interview and access their health records. For those who self-recruited by contacting the study team, we obtained verbal consent to access health records, and the study team confirmed eligibility for the qualitative interviews (i.e., receipt of the MCT’s services in the previous six months). We offered eligible respondents the option of in-person or virtual interviews in English or Spanish. Finally, we obtained all respondents’ demographic and service use information from electronic health records.

We conducted twenty interviews in San Francisco, California, from September 22, 2021, to March 29, 2022. Two interviews were conducted by phone, and the other 18 were in-person in shelters, crisis stabilization units, and public settings. The mean interview length was 51 min. Interviews were audio-recorded for transcription and then de-identified prior to analysis. The University of California, San Francisco Internal Review Board approved all study procedures (IRB #20-32693).

### Semi-structured interviews

We conducted interviews using an iteratively developed semi-structured guide that began with a set of a priori questions based on the MCT’s intended functions. Subsequently, we shared the guide with various subject matter experts, program partners and people with lived experience receiving crisis services. The initial guide centered on MCT’s accessibility, intervention, and assessment; facilitators to post-crisis linkage to care and barriers to subsequent services; and baseline engagement in health care and housing systems. We used open-ended questions and follow-up prompts to elucidate the respondent’s perception of the encounter and the MCT’s role in their broader experiences of homelessness, mental illness, and substance use. We periodically revised the interview guide during data collection based on themes emerging from early interview experiences to ensure that the interviews captured the most salient themes expressed by the respondents. After completing nine interviews, the final iteration of the interview guides incorporated questions exploring how respondents defined their long-term goals and how the MCT and similar programs facilitated the attainment of their goals [see Additional file 1]. We reviewed newly emerging themes during weekly meetings, and the team agreed that by the twentieth interview there were not additional themes emerging and thus saturation had been reached, which is consistent with other qualitative studies [15].

### Data analysis

Similar to developing the semi-structured interview guide, we used an iterative process to create a thematic analysis codebook [see Additional file 2]. The codebook included descriptive codes related to the content of the quotations, as well as descriptive codes related to an MCT recipient’s perceived goals, barriers, and facilitators. Goals were defined as any mention of the respondent’s short- and long-term objectives (e.g., financial independence). Barriers signified any mention of obstacles or perceived limiters to attaining their goals (e.g., resource availability). Facilitators captured any mention of assistance or perceived supporters to attaining their goals (e.g., receiving information about services).

A total of six researchers analyzed the transcripts using Atlas.ti v9 [16]. At least two researchers coded each transcript and discussed salient themes using a deductive approach based on Braun and Clarke’s “theoretical” thematic analysis [17]. Our team reflected on the a priori themes, which prompted recognizing “latent themes,” such as discrepancies between the MCT program’s stated objectives and the respondents’ self-reported goals. Each transcript was presented at weekly qualitative team meetings to reach a consensus about the identified latent themes. Once the study team completed coding the transcripts, one of the two original coders used the final codebook version to review their previously coded transcripts to ensure we captured all potentially relevant themes and resolved any remaining discrepancies.

Once the general themes emerged, we developed a conceptual model to help organize patterns in the data and better understand the needs, goals, and experiences of the study participants. We based this model on the Social-Ecological Model, which uses a set of nested levels organized by proximity to the individual [18–20]:

- Person-level (e.g., social development, health history, individual attributes, traumatic experiences);
- Relationship-level (e.g., family, friends, neighbors, coworkers, individual service providers, social circles).
- Community/Organization level (e.g., service provider organizations, built environment, social milieu, neighborhood); and.
- Society/Policy-level (e.g., laws and regulations, funding priorities, cultural norms, structural inequities).

**Fig. 1:**
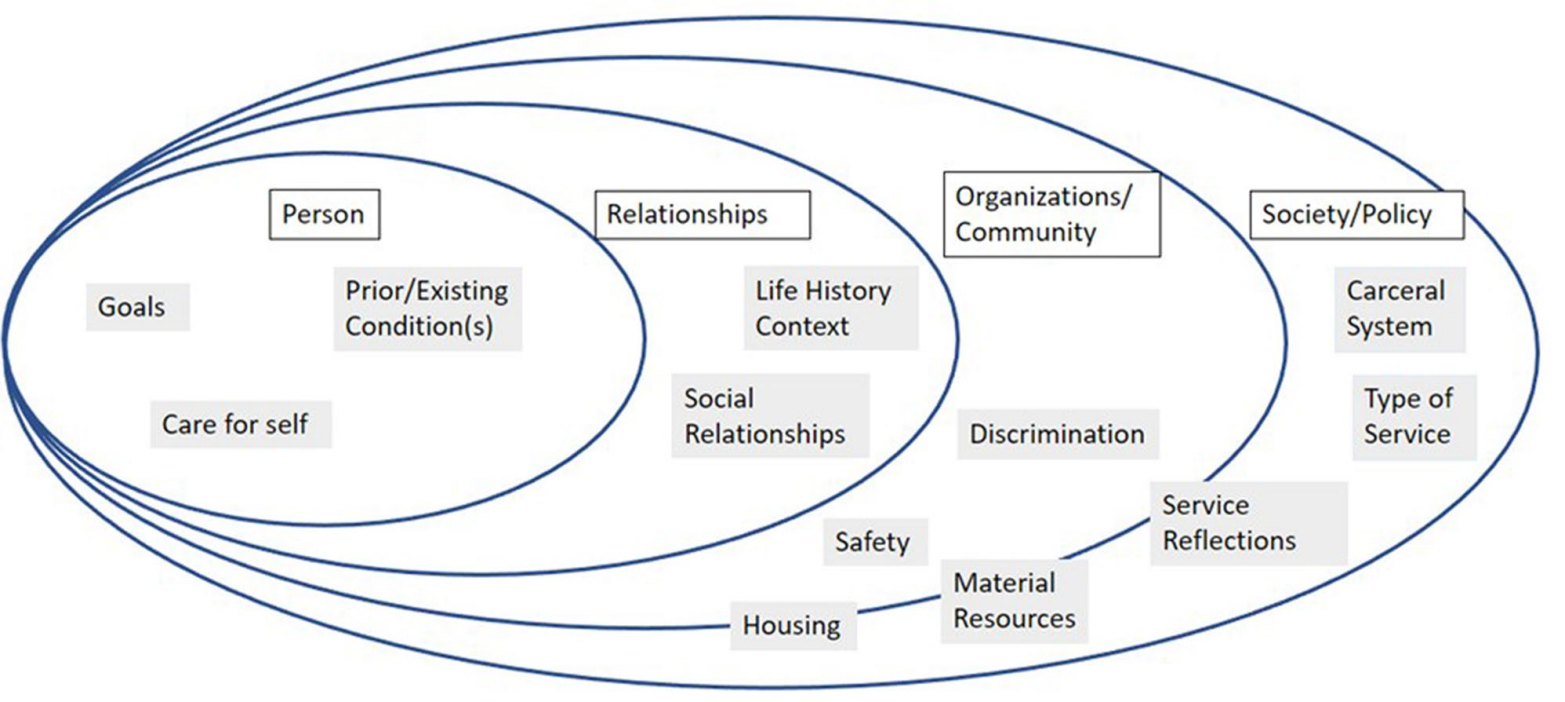
Code groups mapped onto Social-Ecological model

We reviewed and organized our codebook by Social-Ecological levels (Fig. 1). We then categorized codes and themes by factors that affect the attainment of service recipients’ goals across Social-Ecological levels, noting which were reasonably within the scope of the MCT and which were beyond their realm of influence.

## Results

The study team interviewed twenty adults. Respondent ages ranged from 38 to 52 years (median = 43), and there were more men in the sample (75%) than women (25%) (Table 1). Interview participants self-identified their race and ethnicity as 35% Black, 10% White, 20% Hispanic/Latino/a, and 5% Asian, with 30% unreported. The number of days between the MCT encounter and the qualitative interview ranged from 10 to 32 (median = 19).

**Table 1:** Respondent demographics and descriptions at time of the encounter (*N* = 20)

Demographics at the time of the encounter	*N* (%)
Number of days between index MCT encounter and interview	19 (10–32)
Median Age	43 (38–52)
**Age**	
25–34	3 (15%)
35–44	8 (40%)
45–54	5 (25%)
55–64	4 (20%)
**Gender identity**	
Cisgender Female	5 (25%)
Cisgender Male	15 (75%)
**Sex assigned at birth**	
Male	15 (75%)
Female	5 (25%)
**Sexual orientation**	
Straight	12 (60%)
Bisexual	2 (10%)
Lesbian or Gay	2 (10%)
Don’t know	2 (10%)
Missing	2 (10%)
**Race/ethnicity**	
Black/AA	7 (35%)
Hispanic/Latinx	4 (20%)
White	2 (10%)
Asian Pacific Islander	1 ( 5%)
Unknown/No Entry/Missing	6 (30%)
**Primary language**	
English	14 (70%)
Unknown	6 (30%)
Clinical Factors at the time of the encounter	N (%)
**Legal status**	
Voluntary	19 (95%)
Temporary Involuntary	1 ( 5%)
**Suicidality risk during MCT encounter**	
Yes	2 (10%)
No	18 (90%)
**Past-year crisis care service use**	
25th percentile (1 episode)	9 (45%)
50th percentile (2–3 episodes)	3 (15%)
75th percentile (4–10 episodes)	7 (35%)
100th percentile (11 or more episodes)	1 (5%)
**Past-year routine care service use**	
25th -50th percentile (0 episodes)	9 (45%)
75th percentile (1–2 episodes)	3 (15%)
100th percentile (3 or more episodes)	8 (40%)
**Past-year jail entry**	
Yes	1 (5%)
No	19 (95%)
**Received housing assessment before MCT encounter**	
Yes	8 (40%)
No	12 (60%)
**On housing list before MCT encounter**	
Yes	6 (30%)
No	14 (70%)
**Past-year homelessness**	
Yes	19 (95%)
No	1 (5%)

### Person-level

Themes described by MCT recipients that aligned with the innermost level of the Social-Ecological Model included factors intrinsic to the individual such as social development, health history, personal attributes, and experiences of traumatic events and other significant life events [see Additional file 3]. While some respondents mentioned having urgent behavioral health needs at the time of the MCT encounter, a small subset of respondents did not describe the reason for the MCT encounter as a “crisis.” Many mentioned that their emotional state during the encounter was unremarkable, and some could not explain why MCT had arrived. Those who defined their experience relating to a behavioral health crisis found that the MCT was successful in helping address their in-the-moment needs, like food, water, shelter, or blankets. For example, one respondent described the impact of receiving water and warm food during a crisis:

Lots of time, like the police or EMTs, the last thing – they can’t give you water. They’re dealing with life and death, you know? So then [the MCT] said, “Would you like some water?” And I said “Yes,” so they said, “Okay, I’ll be right back”… They got me some nice things of water… Which is good when you’re really thirsty and… then “Yeah, we’ve got some snacks, we’ve got some things, in – in our car. Is there anything – are you hungry or is there anything you’d like?” “Yes. Anything you’ve got, give it.” I was starving. And [my boyfriend] and I, yeah, we’d come down, we hadn’t eaten, we were freezing. So they brough snacks and this hot meal.

Additionally, respondents explained that interactions with the MCT and the dedicated MCT follow-up case managers often bolstered respondents’ motivation to engage with healthcare and housing services. The MCT’s trauma-informed approach centered on the respondents’ crisis needs and preferences, which many said was absent from other crisis services utilized previously. For example, one respondent explained the importance of the team’s collaborative approach:

All three of them, the EMT, the peer counselor, and the provider, the demeanor was, you know, hey, more like brainstorm. Like, how are you? What can we do? What – do you need anything? And, actually, they didn’t say what can we do. They said, would you like some water; you know? That was a big deal.

Respondents described numerous person-level factors affecting their ability to achieve their goals that were outside the MCT’s scope (Table 2). Some reported a variety of chronic mental health, substance use, and medical problems interfering with daily functioning, which intervention by an MCT could do little to ameliorate given the team’s primary focus on emergency crisis services. Many respondents described personal histories of traumatic interpersonal experiences and insufficient emotional support, which understandably may have left them vulnerable to crisis, distrustful of service providers, and lacking effective self-care and coping skills. In a resource-deprived scenario, these personal attributes could be impede service engagement, as described by the following respondent:

**Table 2:** Factors that affect attainment of service recipients’ needs and goals across Social-Ecological levels

	Person	Relationships	Organizations/ Community	Society/ Policy
Definitions of social-ecological model	Social development, health history, individual attributes, traumatic experiences	Family, friends, neighbors, coworkers, service providers (individuals), social circles	Service providers (organizations), built environment, social milieu, neighborhood	Laws and regulations, funding priorities, cultural norms, structural inequities
Factors WITHIN MCT’s scope that affect attainment of service recipients’ goals	Mental health, substance use crisis Experiences of poor health Motivation to engage with services Food, water, blankets Access to phone/email	Emotional connectedness to providers and peers Direct relationships with MCT team members and MCT follow-up Concerns about leaving loved ones to enter treatment or housing	Access to trauma-informed healthcare linkages Access to short-term shelter Short-term safety in the community Respectful interaction with first responders Transportation to needed services Basic necessities like food and water Enrollment in social services (social security insurance, general assistance, etc.) Emotional support from providers and peers	Navigation and coordination to link to a fragmented service system Reduced likelihood of jail entry Modeling how culturally congruent care can support engagement

When you have chronic pain and people don’t know it, they can’t see it. Only you feel it. So, for me it was like I felt like kind of like when I was telling [the social worker], can we get a taxi, and she was just like, “No, we’ll walk to the next one, and then we’ll walk to that one, and then you can get a taxi for the last one.” And I was just like, I wished she would have known. This is my disability, is degenerative disk disease and my mental health, depression, anxiety, PTSD, and schizophrenia. So, for me, if my pain gets angry and the voices are just telling me to kill myself on top of it, and it’s just like, dude, I can’t deal with this stuff. It’s too much.

### Relationship-level

The Social-Ecological Model acknowledges that a person’s relationships with family, friends, and others in someone’s immediate social environment can provide vital support and potential roadblocks to achieving life goals. For some, increased social contact was an important goal, including reconciliation with family. More generally, respondents identified several significant relationship-level factors influenced by the MCT intervention. Many described their relationships with individual MCT and follow-up staff as supportive and motivating. One respondent both underscored the importance of relationships in their life and expressed feelings of closeness toward the MCT:

What’s more important is that – since I’ve been married for 13 years – or a little over 13 years now – I’m just – what’s more important is family. The most important values is family, having my home. Even though my family and her family doesn’t want me anymore, I have my own, right here, in this neighborhood. The Crisis Team – I consider them as family. Whoever comes into my life and treats me good, I consider them as family. Even though we just met. Even though I just met the person, I consider them as family.

Others highlighted how the MCT placed them in treatment programs where they enjoyed supportive relationships with staff and peers. In one case, the respondent described how her aversion to being separated from her partner discouraged her from accepting temporary housing and other resources until the MCT reassured her that they would also help her partner:

They know my name and a life. It’s not the people that were camped over there in the alley. Yeah, they know our name. And that means a lot. And whenever she calls to help me do something, she also helps my husband do something. So, it’s not like she doesn’t play, “Well, I’ll come help you and we’ll leave him out there.” Even if we’re having problems. “Don’t’ worry about what your problem is. I’m helping him.

Respondents described numerous relationship-level factors influencing goal attainment that were outside the scope of the MCT. Many described chronic social isolation as causing poor self-care, ineffective coping, and desperation. Similarly, many described the acute loss of emotional support from relationships as a critical precipitant of mental health and substance use problems. Many respondents relied on financial and housing support from loved ones, making them particularly vulnerable when these relationships were lost. Interpersonal relationships were also described as important sources of both motivation and discouragement to seek services and potential triggers for substance use relapse. Some respondents highlighted the importance of social contacts for facilitating access to employment and services. Finally, a few respondents described how their caregiving responsibilities interfered with their ability to pursue other goals.

### Organization/community-level

The next level of the Social-Ecological Model concerns community-wide factors that influence a person’s ability to achieve their goals, including the built environment, local social norms, and service organizations (including an MCT). Respondents described the MCT as influencing several factors at this level. The importance of access to trauma-informed healthcare services emerged as a recurrent theme in our interviews, and respondents consistently praised MCT staff for their warmth, emotional support, and respect for autonomy. One respondent expressed, “They showed up like that. They gave me the opportunity to have a choice. At the time, I didn’t have a choice. But where I was at on the ground.”

More downstream, some respondents appraised the MCT as successfully linking respondents to crisis stabilization programs and temporary housing resources, promoting short-term safety. Several respondents shared their perception that the MCT had more access to these beds than other outreach services. Follow-up case managers were described as particularly effective, helping respondents access identification cards, entitlements, and assessments for housing eligibility by meeting them in the community and sometimes assisting with transportation. On the other hand, respondents identified many more critical factors outside the scope of the MCT. Most prominently, respondents noted that the MCT had limited ability to connect them with long-term housing, providing temporary relief from the challenges of housing instability. For example, one respondent expressed frustration that the MCT lacked effectiveness in linking them to housing:

Oh yeah, [the MCT] lied to me. They told me that [MCT follow-up case managers] could help me with housing. And then [the case managers] tells me that’s not really what they do. They ran me in circles. They gave me answers that would hold me off until they could get around and delay me later. And eventually, when I got so impatient and tired of waiting for someone to give me a real answer, then they gave me another BS answer and sent me in circles.

Additional barriers to achieving one’s goals included the ever-present risk of bodily harm or theft of possessions, triggering exposures to others’ substance use, and discrimination from community members. Geography appeared to play an important role: many respondents, originally from other cities, described the emotional difficulty of being geographically separated from supportive relationships. Also, some explained that the abundance of desired service providers in neighborhoods they perceived as unsafe made them reluctant to seek help, while often avoided those areas altogether. Several respondents explained that services attempting to address housing instability were ineffective and, at times, had safety concerns that compromised their well-being. For example, one respondent expressed experiences that left them ambivalent about utilizing overnight shelters:

I mean you’re usually out during the day, and it’s really uncomfortable. So, you want to be high, because it’s miserable. And then you’re going back to the shelter. It’s not like really somewhere to go, it’s just somewhere to be out of the elements… Being in shelters is very difficult. And it wasn’t easy. I ended up relapsing in there. And I was using drugs pretty much the duration of my stay there.

### Society/policy-level

The final level in the Social-Ecological Model broadly captures aspects of life regulated by policies and laws as well as social norms. Respondents perceived the MCT and follow-up case managers as having some ability, if limited, to address goal attainment barriers at this level. The follow-up case management did provide some help with navigating the fragmented social service system. Additionally, having the MCT respond to crises instead of or alongside law enforcement was seen by some respondents as helping them avoid incarceration. One respondent considered how the MCT could have produced alternative outcomes to crises previously responded to by law enforcement, saying that:

[W]ell the police wouldn’t had to have come, which makes my anxiety even worse. You know I would’ve just – it would’ve just … They would’ve been able to tell them like this is what it is, you know, like this – she’s having an episode right now. She doesn’t need jail time. She needs to, you know, be treated for her mental, you know.

Most of the factors described by respondents at this level appeared outside the scope of the MCT. Respondents frequently cited inadequate quality, capacity, and general fragmentation in the healthcare and social services systems as barriers to achieving life goals. Several respondents expressed frustration with restrictive and opaque eligibility requirements for entitlements and housing services, which sometimes led them to relive past traumatic experiences in completing assessments to prove their eligibility. Law enforcement policies had a mix of effects on safety experiences; some respondents identified police as helping them secure their possessions and reduce exposure to violence. In contrast, others described law enforcement as not believing them or escalating their distress when in crisis. One respondent recounted the importance of alternatives to law enforcement or emergency medical response:

[The MCT] was the most important, most wonderful thing that I’ve seen. Way different than an ambulance. Way different than the police. Police just stands there and tells you what’s wrong. And they go through your background, and they sit there and chat. But they just look at you, they don’t talk to you like the [crisis] team was aware. They was telling me how you doing? If you want some water. What are you hearing? What are your symptoms? And they were there.

A subset of respondents described a wide range of experiences of discrimination based on race, housing status, and behavioral health conditions that impacted their ability to feel safe and meet their needs. Of those who reported experiencing discrimination, the most common theme was discrimination based on the respondent’s appearance. Some reported that the community was unwelcoming towards those who appeared unhoused (e.g., dirty clothes and poor hygiene). However, others reported that people withheld resources from them because they did not have visible signs of homelessness. One respondent illustrated this perceived differential treatment, saying:

They’re about, “Oh, your clothes are clean. You don’t look like you’re outside.” Well, I choose to use the water, because I don’t have other issues bothering me today. You find me on another day, and I might be all dirty and stuff. Don’t do that. That’s stereotyping and stuff like that. We all need help the same way. You just got me on a different day.

## Discussion

After receiving services from this novel program aimed at engaging PEH in crisis, service recipients described a preference for the MCT over other traditional responders. They further reported that this program helped alleviate short-term challenges that spanned individual, interpersonal, organizational, and societal levels. However, our findings demonstrated that MCT recipients valued and wanted services that could help them attain longer-term goals, which were mostly beyond what the MCT program was able to support. Almost all respondents described goals related to general stability in their lives, most notably housing, as well as employment, family reunification, and reducing drug use. As public health agencies face pressure to respond to the highly visible and challenging social problems related to homelessness, these findings suggest that without also addressing the underlying structural issues faced by PEH, the impacts of an MCT program will likely be limited to short-term improvements such as diversion from law enforcement, engagement with trauma-informed behavioral health specialists, and provision of basic resources.

Although this MCT model was perceived as largely unable to address the longstanding issues facing those impacted by housing instability, our findings suggest that the positive interactions with MCT staff helped restore trust in housing and healthcare systems. The team’s trauma-informed approach and collaborative decision-making helped center the individual’s needs and preferences during the evaluation. Rebuilding a client’s trust with the service system is critical yet often elusive step towards long-term engagement [21]. Although most people considered their housing needs as paramount, these positive connections with providers who are empathetic and well-trained in trauma-informed techniques may lay the foundation for clients to seek future help for behavioral health or substance use problems [22, 23].

While this MCT program aimed to address behavioral health or substance use crises, our findings demonstrated that many respondents did not recall their MCT encounter as occurring during a crisis. This finding raises the possibility that in a 911-dispatched MCT program in which encounters are most often initiated by community members on the person’s behalf, it may not consistently be clear to the bystander or 911 dispatcher whether there is truly a behavioral health crisis. In cases not involving a behavioral health crisis, a non-behavioral health community responder program may be more appropriate to provide social services and engagement, which could free up MCTs to respond only to calls that require evaluation by behavioral health specialists, a limited resource [24]. Furthermore, given the long history of increased interactions between police and PEH due to their visibility in communities and their need to engage in survival behaviors that may contradict social norms [25], requests for MCT services for non-crises could result in avoidable interactions with law enforcement. In order to address the simultaneous priorities to divert non-violent 911 calls from law enforcement and to deliver alternate responses that optimally utilize a constrained behavioral health workforce, there is a need for more clearly established guidelines for triage among MCTs, non-behavioral health community responders, and law enforcement, with referral pathways among teams if needed.

While many MCT recipients reported that they did not have challenges related to behavioral health or substance use, when it was mentioned, many identified their unstable housing as a primary catalyst for the onset or exacerbation of these challenges. This finding is consistent with literature showing increased rates of behavioral health or substance use conditions among PEH [7, 17]. Homelessness is a key driver of the disproportionate burden of disease and will not be solved by a purely behavioral health response [26].

Another prominent theme was the impact of social relationships on the lives of PEH, both positive and negative. MCT recipient perspectives aligned with previous research that describes PEH as feeling rejected or burdensome, which could lead to psychological distress and even suicidality [27]. Furthermore, PEH experience higher rates of social isolation due to being compelled to violate social norms for the sake of survival, which may include highly visible and stigmatized behaviors (e.g., sleeping on the streets, missing routine hygiene, etc.) [28]. Conversely, some respondents described social support as a protective factor in their community. This finding highlights the importance of programs aimed at reconnecting PEH with existing social relationships or establishing new ones for long-term well-being.

### Limitations

This study had several limitations that should be considered when interpreting findings. Despite efforts to recruit all recipients regardless of post-crisis care engagement, due to the challenge of locating specific individuals after a MCT encounter, recruitment relied on the follow-up team, which may have biased the study sample towards service recipients who were most engaged with the system. Recall bias and desirability bias in qualitative interviews may have influenced the willingness of respondents to describe accurate or negative experiences, so the research team attempted to engage with participants as near to the crisis event as possible while using supportive interview techniques. Finally, these findings are based on a single program and may not generalize to other regions, though the themes observed in this urban area may be similar to other localities confronting similar challenges.

## Conclusion

The pervasive and cyclical nature of housing instability is rooted in multiple complex factors that span every level of the social-ecological model described above. Based on the experiences of PEH who received a MCT intervention, our study found that these challenges cannot be fully mitigated by an intervention designed to be a single encounter, with referrals to follow-up services. However, the significant perceived benefits of these MCT interactions related to the way needs were addressed, to trust-building with MCT responders, and to avoidance of unnecessary law enforcement encounters during crisis episodes that do not present a risk of violence. Programs aimed at addressing behavioral health crisis among PEH will likely have limited impact if implemented in isolation and will be better positioned to meaningfully improve the lives of PEH when paired with solutions that can more fully address the barriers people face to achieving their goals in both the short and long term. Program administrators, policymakers, and elected officials should acknowledge these limitations and work together to direct future resources accordingly.

### Electronic supplementary material

Below is the link to the electronic supplementary material.

Supplementary Material 1

Supplementary Material 2

Supplementary Material 3

## Data Availability

The datasets used and analyzed during the current study are available from the corresponding author on reasonable request.

## Abbreviations

MCT: Mobile crisis seam
PEH: People experiencing homelessness
SCRT: Street Crisis Response Team
