# Supplementary material for: “They made me feel like I mattered”: a qualitative study of how mobile crisis teams can support people experiencing homelessness
Source: BMC Public Health. 2024 Aug 12;24:2183. doi: 10.1186/s12889-024-19596-2 (PMC11320767; doi:10.1186/s12889-024-19596-2)
Supplement: Supplementary file 1 — Supplementary Material 1 [file 12889_2024_19596_MOESM1_ESM.docx]

**Additional File 1. Interview guide Version 8.**

**MCT Experience**

- 1. How well do you remember what happened when you met with the MCT on [encounter date]? Tell us a little bit about what you remember.
  2. What was that experience like?
  3. What happened after you met with them?

**Pre-MCT Engagement**

- 1. If you can remember, tell us what your life was like the month or so leading up to that day.
  2. What kinds of things, if anything, were you doing to take care of yourself and your health before that day?
  3. What, if anything, did you know about the MCT before that day?
  4. Before that day, were there times in your life when you needed urgent help or support for your mental health? How do you know you needed help and what, if anything, did you do to resolve the crisis?

**Post-Crisis Linkage**

- 1. Tell us about how your life has been in the days and weeks since you met with the MCT.
  2. What kinds of things are you doing now to take care of yourself and your health?
  3. What has your experience been like with the MCT follow-up case managers?

**Final Questions**

- 1. Tell us a little bit about what is important to you in your life. What are some of your goals? What kinds of things help you achieve these goals? What kinds of things get in the way?
  2. Do you think the MCT has contributed to you getting close or farther from those goals? How?
  3. In an of the experiences we talked about, what ways, if any, have you been treated differently because of your race or ethnicity? How about mental health condition or substance use? What about housing status? How about any other personal characteristics?
  4. Is there anything else you want to share that you think would help us understand your experience better?
