# Supplementary material for: “They made me feel like I mattered”: a qualitative study of how mobile crisis teams can support people experiencing homelessness
Source: BMC Public Health. 2024 Aug 12;24:2183. doi: 10.1186/s12889-024-19596-2 (PMC11320767; doi:10.1186/s12889-024-19596-2)
Supplement: Supplementary file 2 — Supplementary Material 2 [file 12889_2024_19596_MOESM2_ESM.docx]

**Additional File 2. Qualitative codebook Version 9.**

| Code group name | Sub-code name(s) | Code group definition |
| --- | --- | --- |
| Access | 1. Availability of resources 2. Awareness of resources 3. Timeliness of resources 4. COVID-related disruptions   Navigation of resources/systems | Mention of specific aspects related to barriers or facilitators to accessing treatment, housing, social services, and other supportive resources. |
| Barriers to care | No sub-codes (used as an intersecting code across other themes) | A single utility code that almost always co-occurred with “Access” codes. Mention of experiencing challenges when accessing treatment, housing, social services, and other supportive resources. Used for instances in which people talk generally about barriers and in instances with specific barriers are named (e.g., availability). |
| Carceral system | 1. Law enforcement 2. Jail/Carceral system | Mention of an experience with various components of the carceral system, jail, and law enforcement. |
| Care for self | 1. Medication(s) 2. Not medication(s) | Mention of specific ways people care or want to care for themselves that are not considered a service or program. |
| Discrimination | 1. Race/Ethnicity 2. Appearance 3. Mental health condition 4. Physical health condition 5. Language 6. Education or financial status 7. Substance use 8. Housing status 9. Gender identity 10. Sexual orientation and/or behaviors 11. Legal status 12. Other | Mention of differential treatment due to an inherent (e.g., race/ethnicity) or chosen (e.g., appearance) personal characteristic. |
| Facilitators to care | No sub-codes (used as an intersecting code across other themes) | A single utility code that almost always co-occurred with “Access” codes. Mention of experiencing support when accessing treatment, housing, social services, or other supportive resources. Used for instances in which people talk generally about facilitators and in instances with specific facilitators are named (e.g., availability). |
| Goals | No sub-codes (used as an intersecting code across other themes) | A single hybrid utility/descriptive code to flag when a respondent speaks about their goals, motivators, desires, etc. |
| Housing | 1. Housing services 2. Overnight shelter 3. Instability 4. Street | Mention of housing, housing services, shelters, programs, and different aspects of obtaining and maintaining a stable indoor living space intended for human use. |
| Life history context | 1. Child/Adulthood history 2. External/Environmental factors | Mention of past events that impacted the respondent in the years before the MCT encounter. Use this to flag childhood experiences, trauma, abuse, developmental history, neighborhood, adolescent discrimination, family problems, etc. |
| Material resources | 1. Phone 2. Money 3. Food 4. Employment 5. Other | Mention of physical objects the respondent uses, wants, or needs. Employment is included because it often facilitates or impedes attainment of material resources. |
| Prior/Existing condition(s) | 1. Chronic/Complex 2. Mental health 3. Physical health 4. Substance use | Mention of previous or existing conditions related to mental health, physical health, substance use, and chronic/complex conditions. This can refer to specific conditions (e.g., depression) or symptoms of a condition (e.g., hearing voices). |
| Safety | 1. Physical safety 2. Material safety 3. Emotional safety 4. Systemic safety 5. Sexual safety | Compromise/Prevention/Alleviation of physical, material, psychological, or emotional harm. Includes use for flagging active or perceived threats to safety. |
| MCT | 1. Reason for encounter 2. Emotional state 3. Pre-existing impressions 4. Team members 5. MCT follow-up care 6. Post-MCT outcomes 7. Respondent crisis definition | Mention of how a respondent perceived the MCT and how the respondent experienced different components of an MCT encounter including perceived reason for encounter, definition of a crisis, feelings during the encounter and mention of the MCT follow-up case managers. |
| Service Reflections | 1. Reason for non-MCT service engagement 2. Trust in service/support providers 3. Motivation to get help/support 4. Ambivalence about services 5. Effectiveness of services (i.e., desirability) 6. Autonomy 7. Humanization/Validation | Mention of feelings, experiences, opinions, and impressions about services from the respondent and how it relates to engagement with services. |
| Social relationships | 1. Social conflict 2. Isolation 3. Social support/healing 4. Change in social connection | The immediate social environment of the respondent or mention of relevant social contexts, including social support or conflict. Excludes experiences with systems or providers. |
| Type of service | 1. Emergency/Crisis/Inpatient service 2. Physical health routine care 3. Mental health routine care 4. Social service 5. Substance use routine care | Mention of any non-MCT services. |
