# Supplementary material for: “They made me feel like I mattered”: a qualitative study of how mobile crisis teams can support people experiencing homelessness
Source: BMC Public Health. 2024 Aug 12;24:2183. doi: 10.1186/s12889-024-19596-2 (PMC11320767; doi:10.1186/s12889-024-19596-2)
Supplement: Supplementary file 3 — Supplementary Material 3 [file 12889_2024_19596_MOESM3_ESM.docx]

**Additional File 3. Selected quotations organized by code and social-ecological levels.**

| Theme | Quotation |
| --- | --- |
| Person-Level | |
| Prior/ Existing Condition(s) | Everything's like new over again. Because I've been out there stuck in a rut for so long, everything comes new every day. Because out there, the mindset is just so ... I don't know, it's like because I ask God to just keep my mind, protect my mind.  So, I kind of like put it somewhere else. I don't know if that even makes any sense. But it's like out there you've got to be aware of certain things. I ain't got time to think about no doctor. I ain't got time to think about no survey. I ain't got time to think about lunch. I'm worried about where I'm going to lay down tonight, you know? So, everything comes new. And I'm almost kid-like. It's like, oh, I get to go see the doctor today! I'm like, yeah, let's draw some blood! Because now I'm taking the hep C treatment. Wherefore when I was out on the street I didn't take it. I'm on my fifth week of hep C treatment. Like I'm just getting back control. |
| Prior/ Existing Condition(s) | When you have chronic pain and people don't know it, they can't see it. Only you feel it. So, for me it was like I felt like kind of like when I was telling [the social worker], can we get a taxi, and she was just like, "No, we'll walk to the next one, and then we'll walk to that one, and then you can get a taxi for the last one." And I was just like, I wished she would have known. This is my disability, is degenerative disk disease and my mental health, depression, anxiety, PTSD, and schizophrenia. So, for me, if my pain gets angry and the voices are just telling me to kill myself on top of it, and it's just like, dude, I can't deal with this stuff. It's too much. |
| Prior/ Existing Condition(s) | And I'm still not ready. You know something? I'm still not ready to let go of the drugs. I want to, I have to, I need to, but I don't want to 100 percent let go of that feeling that I get when I get high. That euphoria. So I'm into harm reduction, but – and I am so for harm reduction, you guys, it will save lives. I think my boyfriend wouldn't have died if he had it. If people would have been realistic with him. I want me to stop. I want people I love never to touch it again. But it doesn't always work that way. |
| Goals | Well, you know, I mean it would make a lot different if I got a place to stay. That's one that would make a lot of difference. Really if I got a place to go that, you know, I could really be there. Like the DMV or the SSI.  Or even see a psychiatrist, you know, I mean or even for myself, you know, I mean I would like to go to the park and play my guitar, you know. And just – so, it's just those kinds of things that just, that I ... That I plan to start doing pretty soon, you know. Hopefully, I get to put this luggage of mine in storage and not worry about it getting lost. And be able to settle myself back to where, you know, settle down to a place or get back to where I'm – to my hometown, you know. |
| Goals | Now, I got a plan. Now I have to set goals and then reach those goals. And then I can set new goals. In the streets, there’s no plan and there’s no goal. |
| Care for Self | And people, you know, like, you're saying were you taking care of yourself, and someone – a lot of us addicts would say, oh, God, I wasn't. I was horrible. Of course, I wasn't. Because that what society tells you. You're not taking care of yourself. Look at you. You were a mess. But deep down, I want to tell you that we do think – we want to take care of ourselves, and we think we are to a certain extent. Especially, you know what? The drug makes us feel like we're really taking care of ourselves. Even though, like I said, crack, heroin, that, but when you – when you feel the feeling the drug gives you, or for me, when I feel the feeling the drug gives me, I feel like I am a self-contained unit. I feel like I'm a self-cleaning oven.  Like, I have, oh, my God, everything is going to be okay. I'll just do this and that and this – like, you could – I've told people you could be in your own pee on Ellis Street and with not a cent to your name, barefoot, you know, sick, you know, just a mess, and you take a hit of crack or whatever your drug is – maybe you're an alcoholic – but for me, you take a hit of crack and all of a sudden, you can handle everything.  This isn't so bad. I'll just get up, I will clean off, I'm going to go get this, I'm going to go ask – access that kind of help, I'm going to do this, you – all of a sudden, you're this adept human being again. And you're going to take care of you and you have the warm sense of, like, security. And then you need another hit. And then you need another. And then another. And then you start getting urgent. And then it change – your addiction changes. |
| Relationship-Level | |
| Life History Context | I want to help myself become a better person. Hell, I'm 64 years old, you know, I don't know how much more time I have left here. I have seven grandchildren, you know, and, I want to be around. … My grandfather passed, and he died in the streets of San Francisco. Some crazy shit like that, you know, I don't want that. I can't deal with that anymore, you know, like I said. |
| Social Relationships | Now the people in the community, that's different. People from the city, they're nice people. Every neighborhood is different. And then we like it that way. |
| Social Relationships | What's more important is that – since I've been married for 13 years – or a little over 13 years now – I'm just – what's more important is family. The most important values is family, having my home. Even though my family and her family doesn't want me anymore, I have my own, right here, in this neighborhood. The Crisis Team – I consider them as family. Whoever comes into my life and treats me good, I consider them as family. Even though we just met. Even though I just met the person, I consider them as family. |
| Social Relationships | I'm glad I might be able to use what I learned, my experiences, to help others. That's the only reason it'll work out. That way it won't be a – my time won't be lost time. |
| Social Relationships | We're like a family. Homeless people on the street are family. They're a family that never leaves you. They will always be there for you and that's there for you. Just like your family, your biological's family there for you, you know.  Some of your family members die, pass on, you know. But your street family will always be there for you too. So, I mean we're all family here. Really, we are. I mean, you know, I pretty much know everybody from history in here from the streets. I do. Just nearly everybody I do. And it's never spoke about or talked about. But it is sometimes. You know it's true. |
| Organization/Community-Level | |
| Discrimination | It is, because they're about, "Oh, your clothes are clean. You don't look like you're outside." Well, I choose to use the water, because I don't have other issues bothering me today. You find me on another day, and I might be all dirty and stuff. Don't do that. That's stereotyping and stuff like that. We all need help the same way. You just got me on a different day. |
| Discrimination | Well, I wasn't getting access to my critical care like I was. But it ... I used to get access to critical ... I used to get access to medical treatment a lot. And then it's like the times is changing. And it's not happening like it used to. They think that I'm just like ... Some people think that I'm like wasting peoples' money. Wasting taxpayers' money. They think I'm wasting peoples' time and money. And I'm not doing that, you know. Because like that man right there in a wheelchair, I'm kind of like disabled like him, you know... I can't really walk too much. And those wheelchairs cost a lot too. |
| Safety | Everything that I have that is valuable, I'll probably not have it anymore. Because I don't have nowhere to put it. |
| MCT follow-up care | They know my name and a life. It's not ‘the people that were camped over there in the alley.’ Yeah, they know our name. And that means a lot. And whenever she calls to help me do something, she also helps my husband do something. So, it's not like she doesn't play, "Well, I'll come help you and we'll leave him out there." Even if we're having problems. "Don't' worry about what your problem is. I'm helping him, too." And I love that. Because they don't leave anybody behind. |
| Housing | And [the MCT team] came out and asked me, “what did I need help with,” and I made it clear that I was trying to get ... I made it clear that I needed permanent housing…that was my main goal - not to just get a shelter, but I need permanent housing. And I'm active, I let them know that I was active on a certain waiting list for subsidized housing. And I needed to directly get a hold of that establishment. So that way they could know my crisis here on the street and help me from there to get a house. Because my housing, I want my housing. |
| Discrimination | I have to prove that I'm sane to be a lot – I mean to survive around these people. You know even with jobs. When they find out you have mental issues, they want to make sure it's not the mental issues that they want in their company, you know?…So, it's always like you have to always prove yourself to somebody. |
| Society/Policy-Level | |
| Access | So, I went down and I took the at-risk survey, and I answered "yes" to every question, domestic abuse, drug abuse, sexual abuse, everything. And they still said I didn't qualify. I'm like, what the fuck? And they're like, "Well, we don’t know. We're not really sure why." And then, a couple days later, one of the supervisors of HYA comes up to me and was like, "Well, you're doing too good to get housing." I'm like, what the hell do you mean? It's the American dream right here to live in the fucking parking lot in a tent? Like really? |
| Access | Everything else I can get. I can find it somehow. The only thing I can't find is a place to set up, rest my head, and feel peace. |
| Carceral System | [W]ell the police wouldn't had to have come, which makes my anxiety even worse. You know I would've just – it would've just ... They would've been able to tell them like this is what it is, you know, like this – she's having an episode right now. She doesn't need jail time. She needs to, you know, be treated for her mental, you know. |
| Housing | What made me to go to the [hospital] – I had two choices young lady. That I would either go to the shelter or to I was going to the hospital ... Okay. Me going to the hospital. I said the hospital would be better because I could be around. I can see trained professionals. A psychologist and a psychiatrist. If I had any medical things, I could deal with them too while I was there.  So, I feel like it's safer. It was a safer bet for me than going into some shelter, running into a bunch of – some fools that were, you know, have some words with me or what have you. And then it's the thing of being in those type of environments that, you know, I felt very unsafe. I feel unsafe in that – I just can't get – I won't be around that type of environment. And that I'm not – let me explain something to you both, yeah. I'm not better than any ... I'm not better than any person. |
| Safety | Everything that I have that is valuable, I'll probably not have it anymore. Because I don't have nowhere to put it. |
| Safety | I went to a – I tried to go to rehab, Walden House, and when I was in Walden House, the first couple of – the first two times kind of felt like jail, so I walked out. And then the third time, these kids were relapsing in my room. And a kid overdosed on fentanyl, so I had to run into the restroom and luckily when I worked for Urban Alchemy, I learned how to use Narcan. And so, I ran in there and I Narcan-ed this kid in the restroom five times and brought him back. |
| Service Reflections | The system is only helping people that already got it… I mean the system's designed that way. |
| Service Reflections | [The hospital workers] got me out of the storm. I ended up back in the storm. It was kind of weird. But they got me out of the storm for a while, but the hospital couldn't keep me. I had hypothermia and stuff. And then once the hypothermia went away, it was like they released me again, so I ended up back out in the cold again. Another storm was coming. Ended up with hypothermia again. |
